# Characterization of integrons among *Escherichia coli* in a region at high incidence of ESBL-EC

**DOI:** 10.12669/pjms.301.4079

**Published:** 2014

**Authors:** Lu-Ming Li, Ming-Yi Wang, Xiao-Yan Yuan, Hong-Jun Wang, Qin Li, YA-Mei Zhu

**Affiliations:** 1 Lu-Ming Li, Ming-Yi Wang, Department of Clinical Lab, Department of Cardiovasology, Weihai Municipal Hospital affiliated to Dalian Medical University, Weihai, Shandong, 264200, PR China.; 2 Xiao-Yan Yuan, Department of Clinical Lab, Weihai Municipal Hospital affiliated to Dalian Medical University, Weihai, Shandong, 264200, PR China.; 3 Hong-Jun Wang, Weihai Municipal Hospital affiliated to Dalian Medical University, Weihai, Shandong, 264200, PR China.; 4 Qin Li, Weihai Municipal Hospital affiliated to Dalian Medical University, Weihai, Shandong, 264200, PR China.; 5 YA-Mei Zhu, Weihai Municipal Hospital affiliated to Dalian Medical University, Weihai, Shandong, 264200, PR China.

**Keywords:** Blood stream infection, ESBL-EC, Integron, Gene cassette.

## Abstract

***Objective*** *: *The aim of study was to investigate the distribution of the integrons in *Escherichia coli* (*E. coli)* isolates, and analyze the possible relationship between the antimicrobial resistance profiles and the integrons.

***Methods*** ***:*** The antimicrobial profiles of 376 *E. coli* strains were analysed by disk diffusion test. The integron genes and variable regions were detected by PCR. Some amplicons were sequenced to determine the gene cassettes style.

***Results*** ***:*** Of 376 isolates, 223 isolates (59.3%) were confirmed as ESBL-EC. Comparison to ESBL-negative *E. coli*, the high rates of resistance to the third and fourth generation of cephalosporins, penicillins and amikacin were found in ESBL-EC. Only class 1 was integron detected in the isolates, and the prevalence of it was 66.5%. It was commonly found in ESBL-EC (77.6%, 173/223), which was higher than that of ESBL-negative* E. coli *(50.3%, 77/153) (*p<0.001*). Six different genes cassettes were detected in this study and were classified into three groups: *dfr17*-*aadA5*, *dfrA12-aadA2 *and* aacA4-CmlA1. *Additionally, more than one gene array harboured in 13.9% isolates of ESBL-EC, while in 9.1% isolates of ESBL-negative* E.coli.*

***Conclusion*** ***:*** The high incidence of ESBL-EC with resistance to multiple antibiotics were detected in the isolates from Blood stream infection (BSI). More resistant gene cassettes in ESBL-EC may partially underlie the high resistance to amikacin, while no relation exists between the high incidence of ESBL-EC and classes 1~ 3 integrons in this region.

## INTRODUCTION

Bloodstream infection (BSI) is associated with major morbidity and mortality, and *Escherichia coli* (*E.coli*) is one of the most comment microorganisms isolated from this infection.^1^ In recent years, the detection rate of extended spectrum β-lactamases (ESBL) in enterobacteriaceae bacteria, specially *Escherichia coli *(*E. coli)* is increasing.^2^ It is well known that ESBL-producing organisms are resistant to multiple unrelated antibiotics^.^^3^ As a result, multiple drug-resistant clinical infections of *E. coli *are now common. Integrons are widespread genetic elements responsible for dissemination of antibiotic resistance among Gram-negative bacteria, being commonly found in plasmids and/or transposons.^4^ Recently integrons have become an important drug-resistance mechanism of *E. coli*.^5^ ESBL-producing* E.coli *(ESBL-EC) carrying integrons will have more drug resistance genes and resistance to many other drugs.

Knowledge of the characterization of integrons among *E. coli* isolated will contribute to assess the phenotypic and genetic characterization of antimicrobial profiles of the organism in BSI. In this study, we investigated classes 1, 2, and 3 integrons associated integrases genes in *E.coli* isolates from blood to evaluate integron characterization and typing of this organism.

## RESULTS

A total of 376 *E. coli *isolates were taken during the study period. Of these, 223 isolates (59.3%) were comfirmed as ESBL-EC. None of isolates were all susceptible to antimicrobial agents, and the phenotypic characterization of antimicrobial profiles are shown in Table-II. Among the isolates, the high rate of resistance to levofloxacin, ciprofloxacin and piperacillin were 59.8%, 64.9% and 84.8% respectively, with the low rate of resistance to imipenem and meropenem (0.5% and 0%). Comparison to ESBL-negative *E. coli*, the high rates of resistance to the third and fourth generation of cephalosporins, penicillins and amikacin were observed in ESBL-EC.

***The Prevalence of classes 1, 2 and 3 integrons status in*** ***E. coli: ***The prevalence of classe 1 integron in *E. coli* isolates was 66.5% (250/376). It was commonly found in ESBL-EC (77.6%, 173/223), which was higher than that of ESBL-negative* E. coli *(50.3%, 77/153) (*p<0.001*). No amplification products were obtained from any of these isolates when the primers specific for intI2 or intI3 were used.

Classe 1 integron was detected on genomic and plasmid DNA in 155 isolates (77%, 155/201) of ESBL-EC, but it was detected on genomic or plasmid DNA in 9 isolates respectively. While Classe 1 integron was haboured on genomic and plasmid DNA in 29 isolates (37.7%, 29/77) of ESBL-negative *E. coli*, most of others (30 isolates) was haboured on plasmid DNA.

***The Characteristic of Class 1 Integron in Isolates: ***Each gene-cassette region in class 1 integrons was amplified to identify characteristic of gene cassette in the isolates. 164 ESBL-EC isolates (94.8%, 164/173) of carrying class 1 integron contained resistant gene cassette, and the other 9 isolates were detected no resistant gene cassette. For ESBL-negative *E. coli*, 56 isolates (72.7%, 56/77) were detected resistant gene cassette.

All these gene cassettes detected were divided into 6 different ones: *dfrA17, aadA5, dfrA12, aadA2, aacA4 and CmlA1*. The proteins encoded by gene cassette may contribute to the resistance of bacteria isolates to trimethoprim (*dfr17 and dfrA12*), aminoglycosides (*aadA5, aadA2, and aacA4*), and chloramphenicol (*CmlA1*).

**Fig.1:**
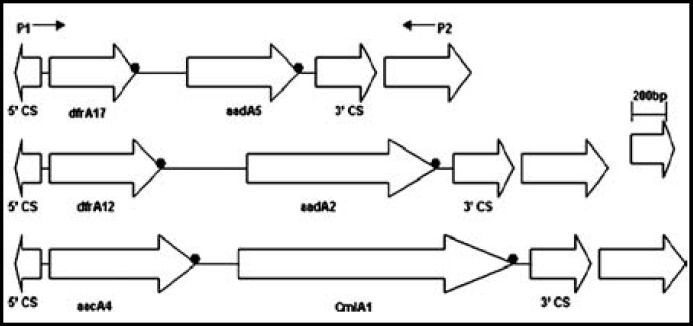
Schematic representation of the genetic elements of Class 1 Integron in *E coli* isolated from BSI.

The integrons were classified into three groups according to the length of amplicons (Fig.1). The *dfr17*-*aadA5* array was the most prevalent in the isolates which lengths most were 1,600bp and the other two arrays were *dfrA12-aadA2, *and* aacA4-CmlA1* which lengths were 2,000bp and 2,400bp, respectively. More than one gene array harboured in 13.9% (24/173) isolates of ESBL-EC, while in 9.1% (7/77) isolates of ESBL-negative* E.coli.*

## DISCUSSION

Despite the great advances in medical science in the past century, BSI remains a growing public health concern worldwide. *E. coli* is one of the most common pathogens for this kind infection. Over the past few years, a significant increase in the number of ESBL-EC-associated BSI is being reported in several parts of the world.^1^^,^^8^ In our study, about 60% *E. coli* isolates from BSI were ESBL-EC, which is higher than other reports (7.3% in Hong Kong, 1.5–16.7% in Taiwan).^9^^,^^10^

**Table-I:** Oligonucleotide primers used in the PCR assay

*Primer*	*Nucleotide sequence (5’-3’)*	*PCR target*	*Expected size (bp)*
IntI1F	ACGAGCGCAAGGTTTCGGT		
IntI1R	GAAAGGTCTGGTCATACATG	Class 1 integrase gene	565
IntI2F	GTGCAACGCATTTTGCAGG		
IntI2R	CAACGGAGTCATGCAGATG	Class 2 integrase gene	403
IntI3F	CATTTGTGTTGTGGACGGC		
IntI3R	GACAGATACGTGTTTGGCAA	Class 3 integrase gene	717
5'-CS	GGCATCCAAGCAGCAAG		
3'-CS	AAGCAGACTTGACCTGAT	Variable region of integrons	Uncertain

**Table-II:** Antimicrobial resistance for 376 *E. coli* isolated from BSI (%).

Antimicrobials*	*AMZ*	*AK*	*SCF*	*CIP*	*CRO*	*FOX*	*IMP*	*LEV*	*MEM*	*PRL*	*CTX*	*CAZ*	*TZP*	*FEP*
ESBL-EC (223)	26.5	52.0	12.6	78.0	100	26.0	0.9	78.0	0	100	100	60.0	26.0	60.5
ESBL-negative E. coli	13.1	3.9	0	45.8	12.4	8.5	0	33.3	0	62.7	16.3	3.9	0	0
Total	21.0	32.4	7.4	64.9	64.4	18.9	0.5	59.8	0	84.8	66.0	37.2	15.4	35.9

^*^Abbreviations in the table: Amoxicillin–clavulanic acid (AMZ); Amikacin (AK); Cefoperazone/sulbactam (SCF); Ciprofloxacin (CIP); Ceftriaxone (CRO); Cefoxitin (FOX); Imipenem (IMP); Levofloxacin (LEV); Meropenem (MEM); Piperacillin (PRL); Cefotaxime (CTX); Ceftazidime(CAZ); Piperacillin*-*Tazobactam (TZP); Cefepime (FEP)

The high rates of resistance to levofloxacin and ciprofloxacin were found in the study, while low rates of resistance to imipenem and meropenem existed in vitro. Carbapenems use has been associated with the low risk of death in cases of serious infections caused by these pathogens.^11^^,^^12^ Thus, these antimicrobials are first line therapy in patients with serious infections. Additionally, the resistance to amikacin was significantly higher in ESBL-EC than in ESBL-negative* E. coli *except to the third and fourth generation of cephalosporins and penicillins.

Integrons had come under observation in 1989 by Stokes for the first time^13^, which could carry more than forty resistance genes. The resistant gene could spread to other bacteria through the integrons, so it is very important to monitor* E. coli *strains isolates from BSI.

We screened the three classes integrons associated integrases genes by PCR, and found only class 1 integron gene was haboured in *E. coli* isolated from BSI. The prevalence of class 1 integron in *E. coli *isolates was in accordance with other’s reports^14^, while the prevalence was higher in ESBL-EC than in ESBL-negative *E.coli. *Additionally, the prevalence of class 1 integron on genomic and plasmid DNA was higher in ESBL-EC. We found six different gene cassettes in class 1 integron and three arrays in the study. These results also showed that the characterization of integrons in bacteria obviously had regional difference.^15^^,^^16^ Although the same resistant gene cassettes and arrays were found on ESBL-EC and ESBL-negative *E.coli*, the carrying rate of gene cassettes in ESBL-EC was higher than that in ESBL-negative* E.coli.*

In conclusion, the high incidence of ESBL-EC with resistance to multiple antibiotics were detected in the isolates from BSI. Comparision to ESBL-negative *E.coli*, more resistant gene cassettes in ESBL-EC may partially underlie the high resistance to amikacin, while no relation exists between the high incidence of ESBL-EC and classes 1~3 integrons in this region.

## Notes

***Disclosure of interest: ***The authors declare that they have no conflicts of interest concerning this article.
